# Supplementary material for: Malignant solid tumor-related spontaneous intracerebral hemorrhage: a propensity score matching study
Source: PeerJ. 2024 Dec 23;12:e18737. doi: 10.7717/peerj.18737 (PMC11670766; doi:10.7717/peerj.18737)
Supplement: Supplemental Information 1 [file peerj-12-18737-s001.doc]

STROBE Statement—checklist of items that should be included in reports of observational studies

|  | Item No | Recommendation | **Reported on Page**  **Number/Line**  **Number** |
| --- | --- | --- | --- |
| **Title and abstract** | 1 | Clinical characteristics and prognostic factors of adult T lymphoblastic lymphoma patients combined with molecular biological characteristics | Page 1/Line 1-2 |
|  |  | (*b*) T-LBL may have its unique molecular biology, with high-frequency mutated genes detected in multiple signaling pathways.Our study showed that mediastinal offenders were more frequent in the Chinese population(70.8%),and the proportion of extranodal invasion was also higher（39.33%）probably related to a poor prognosis.Prognostic grouping combined with molecular biology features can facilitate more accurate identification of high-risk patients and develop better therapeutic strategies with new targeted agents. | Page 1/Line 3-19 |
| Introduction | | |  |
|  | 2 | Exploring the clinical characteristics, molecular biological characteristics and prognostic factors of T-LBL can help to further identify high-risk patients for stratified treatment and seek specific therapeutic targets to improve their efficacy and improve long-term survival. | Page 1/Line 20 to Page 2/Line 21 |
| Objectives | 3 | To analyze the clinical characteristics of T lymphoblastic lymphoma (T-LBL), and explore the prognostic characteristics of adult T-LBL combined with molecular biological characteristics and the prognostic impact of different chemotherapy options on T-LBL. | Page 1/Line 21-23 |
| Methods | | |  |
| Study design | 4 | retrospective analysis | Page 2/Line 1-12 |
| Setting | 5 | 89 patients with T-LBL aged from 18 to 72 years , seen at our Institute from January 2010 to November 2023. | Page 2/Line 13-15 |
| Participants | 6 | Admission requirements for the study included histopathology, histochemistry, immunophenotyping, molecular biology and cytogenetics.Diagnosis and classification were on the basis of the WHO criteria for classification of lymphohematopoietic tissue tumors.  c.LBL was diagnosed when the tumor presented without or only minimal blood and marrow involvement (primitive and naive lymphocyte ratio 25% in bone marrow),the 25% proportion of bone marrow protocells is not included. | Page 2/Line 16-25 |
|  |  |  |  |
| Variables | 7 | Clinical characteristics,Immunophenotype,Gene expression frequency and characteristics,Efficacy and survival analysis | Page 2/Line 26 to Page 3/Line 10 |
| Data sources/ measurement | 8* | According to the efficacy assessment of the lesion invasion site at the initial diagnosis, all bone marrow offenders underwent bone marrow examination in each cycle.Efficacy was assessed in all patients by positron emission computerized tomography(18F-FDG PET/CT) or computerized tomographic scanning(CT) after every two cycles of chemotherapy.Clinical characteristics ，immunophenotype and gene expression frequency were collected. | Page 3/Line 11-20 |
| Bias | 9 | The 25% proportion of bone marrow protocells is not included. | Page 2/Line 31 to Page 3/Line 5 |
| Study size | 10 | Sample size was calculated based on the primary outcome measures | Page 3/Line 6-7 |
| Quantitative variables | 11 | Explain how quantitative variables were handled in the analyses. If applicable, describe which groupings were chosen and why | Page 3/Line 7-10 |
| Statistical methods | 12 | (*a*) Data processing and analyses were done by R version 4.3.0 (2023-04-21), together with Storm Statistical Platform (www.medsta.cn/software). According to the chemotherapy regimen into two groups, the data of the two groups were analyzed with t-test and χ 2 test; the Kaplan-Meier method was used for survival analysis, Cox regression analysis, and the survival rate of the two groups was analyzed by Log rank comparison, P <0.05 was statistically significant. | Page 3/Line 10-19 |

Continued on next page

| Results | | |
| --- | --- | --- |
| Participants | 13* | (a) Report numbers of individuals at each stage of study—eg numbers potentially eligible, examined for eligibility, confirmed eligible, included in the study, completing follow-up, and analysed |
|  |  | (b) Give reasons for non-participation at each stage |
|  |  | (c) Consider use of a flow diagram |
| Descriptive data | 14* | (a) Give characteristics of study participants (eg demographic, clinical, social) and information on exposures and potential confounders |
|  |  | (b) Indicate number of participants with missing data for each variable of interest |
|  |  | (c) *Cohort study*—Summarise follow-up time (eg, average and total amount) |
| Outcome data | 15* | *Cohort study*—Report numbers of outcome events or summary measures over time |
|  |  | *Case-control study—*Report numbers in each exposure category, or summary measures of exposure |
|  |  | *Cross-sectional study—*Report numbers of outcome events or summary measures |
| Main results | 16 | (*a*) Give unadjusted estimates and, if applicable, confounder-adjusted estimates and their precision (eg, 95% confidence interval). Make clear which confounders were adjusted for and why they were included |
|  |  | (*b*) Report category boundaries when continuous variables were categorized |
|  |  | (*c*) If relevant, consider translating estimates of relative risk into absolute risk for a meaningful time period |
| Other analyses | 17 | Report other analyses done—eg analyses of subgroups and interactions, and sensitivity analyses |
| Discussion | | |
| Key results | 18 | Summarise key results with reference to study objectives |
| Limitations | 19 | Discuss limitations of the study, taking into account sources of potential bias or imprecision. Discuss both direction and magnitude of any potential bias |
| Interpretation | 20 | Give a cautious overall interpretation of results considering objectives, limitations, multiplicity of analyses, results from similar studies, and other relevant evidence |
| Generalisability | 21 | Discuss the generalisability (external validity) of the study results |
| Other information | | |
| Funding | 22 | Give the source of funding and the role of the funders for the present study and, if applicable, for the original study on which the present article is based |

*Give information separately for cases and controls in case-control studies and, if applicable, for exposed and unexposed groups in cohort and cross-sectional studies.

**Note:** An Explanation and Elaboration article discusses each checklist item and gives methodological background and published examples of transparent reporting. The STROBE checklist is best used in conjunction with this article (freely available on the Web sites of PLoS Medicine at http://www.plosmedicine.org/, Annals of Internal Medicine at http://www.annals.org/, and Epidemiology at http://www.epidem.com/). Information on the STROBE Initiative is available at www.strobe-statement.org.
