# Supplementary material for: Malignant solid tumor-related spontaneous intracerebral hemorrhage: a propensity score matching study
Source: PeerJ. 2024 Dec 23;12:e18737. doi: 10.7717/peerj.18737 (PMC11670766; doi:10.7717/peerj.18737)
Supplement: Supplemental Information 2 [file peerj-12-18737-s002.zip › Supplementary data/Figures.pptx]

## Slide 1
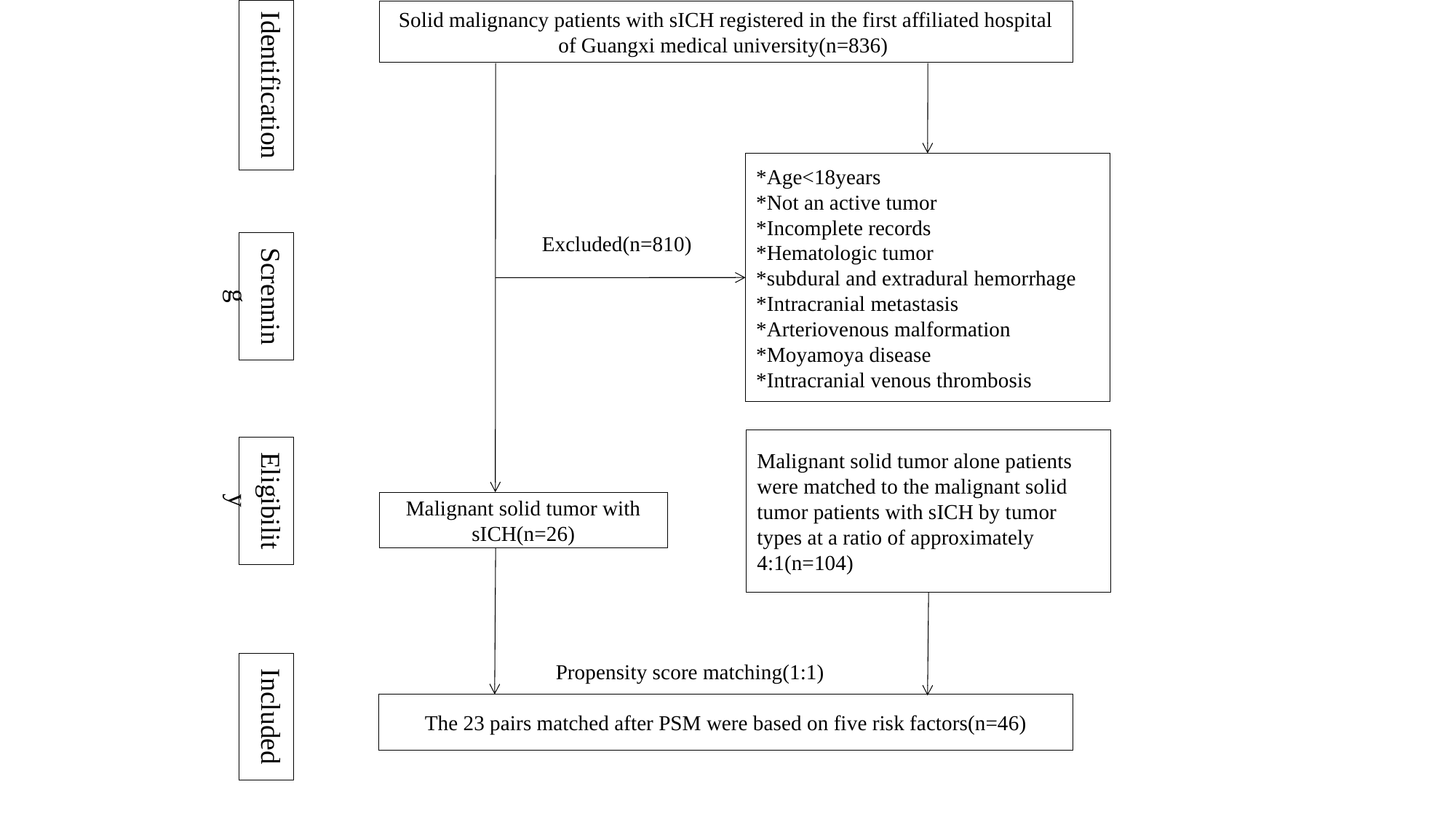

Identification
Solid malignancy patients with sICH registered in the first affiliated hospital of Guangxi medical university(n=836)
*Age<18years
*Not an active tumor
*Incomplete records
*Hematologic tumor
*subdural and extradural hemorrhage
*Intracranial metastasis
*Arteriovenous malformation
*Moyamoya disease
*Intracranial venous thrombosis
Excluded(n=810)
Screnning
Malignant solid tumor alone patients were matched to the malignant solid tumor patients with sICH by tumor types at a ratio of approximately 4:1(n=104)
Eligibility
Malignant solid tumor with
sICH(n=26)
Propensity score matching(1:1)
Included
The 23 pairs matched after PSM were based on five risk factors(n=46)
